# *Urtica pilulifera* leaves extract mitigates cadmium induced hepatotoxicity via modulation of antioxidants, inflammatory markers and Nrf-2 signaling in mice

**DOI:** 10.3389/fmolb.2024.1365440

**Published:** 2024-02-26

**Authors:** Shaimaa Hussein, Abir Ben Bacha, Mona Alonazi, Maha Abdullah Alwaili, Maysa A. Mobasher, Najla Ali Alburae, Abeer A. Banjabi, Karim Samy El-Said

**Affiliations:** ^1^ Department of Pharmacology, College of Pharmacy, Jouf University, Sakaka, Al Jawf, Saudi Arabia; ^2^ Department of Biochemistry, College of Science, King Saud University, Riyadh, Saudi Arabia; ^3^ Department of Biology, College of Science, Princess Nourah bint Abdulrahman University, Riyadh, Saudi Arabia; ^4^ Department of Pathology, Biochemistry Division, College of Medicine, Jouf University, Sakaka, Saudi Arabia; ^5^ Department of Biology, Faculty of Science, King Abdulaziz University, Jeddah, Saudi Arabia; ^6^ Department of Biochemistry, Faculty of Science, King Abdulaziz University, Jeddah, Saudi Arabia; ^7^ Biochemistry Division, Chemistry Department, Faculty of Science, Tanta University, Tanta, Egypt

**Keywords:** *Urtica pilulifera*, antioxidants, nuclear-related factor-2, cadmium, hepatotoxicity

## Abstract

**Introduction:** Cadmium (Cd) is a harmful heavy metal that results in many toxic issues. *Urtica pilulifera* showed potential pharmaceutical applications. This study investigated the possible ameliorative mechanism of *Urtica pilulifera* leaves extract (UPLE) against hepatotoxicity induced by cadmium chloride (CdCl_2_) in mice.

**Methods:** *In vitro* phytochemical screening and the metal-chelating activity of UPLE were ascertained. Four groups of forty male mice were used (*n* = 10) as follows; Group 1 (G1) was a negative control. G2 was injected i.p., with UPLE (100 mg/kg b. wt) daily. G3 was injected i.p., with Cd (5 mg/kg b. wt) daily. G4 was injected with Cd as in G3 and with UPLE as in G2. On day 11, the body weight changes were evaluated, blood, and serum samples were collected for hematological and biochemical assessments. Liver tissues were used for biochemical, molecular, and histopathological investigations.

**Results:** The results showed that UPLE contains promising secondary metabolites that considerably lessen the negative effects of Cd on liver. Furthermore, UPLE inhibited oxidative stress and inflammation; restored antioxidant molecules; and promoted nuclear-related factor-2 (Nrf-2) expression. Also, UPLE improved the histopathological alterations induced by Cd.

**Discussion:** This study explored the beneficial role of UPLE treatment in Cd-induced liver injury through enhancing Nrf-2 signaling and antioxidant enzyme gene expression in the liver of mice. Therefore, UPLE could have valuable implications against hepatotoxicity induced by environmental cadmium exposure. Which can be used as a chelating agent against Cd.

## 1 Introduction

The exponential worsening of environmental problems caused by the rapid development of the human population led to an increase in industrial pollution. Particularly, heavy metals are released into the environment that cause toxicities in certain organs of the human body (Mitra et al., 2022). Cadmium (Cd) is a cytotoxic heavy metal, which raises the possibility of negative consequences on human health, causing hepatorenal dysfunctions, pulmonary edema, testicular damage, and hemopoietic system damage (Genchi et al., 2020). The precise underlying mechanisms responsible for the harmful effects of heavy metal exposure are quite complex, oxidative stress and inflammation are key markers for Cd-induced tissue damage (Das and Al-Naemi, 2019). A previous study reported that cadmium-induced oxidative stress triggers a pro-inflammatory cascade that accounts for the destruction of synaptic branches in the central nervous system (Branca et al., 2020). Furthermore, Cirmi et al. (2021) reported the Cd-induced kidney injury in mice and the role of natural antioxidants in ameliorating oxidative stress via the enhancement of different defense mechanisms.

Natural antioxidants demonstrated potential for tracking down free radicals and neutralizing the harmful effects of heavy metal toxicities (Abu-Khudir et al., 2023). The plant-derived bioactive metabolites have been used as therapeutic agents against heavy metals that cause tissue damage (El-Said et al., 2022). For instance, resveratrol effectively plays a major role in alleviating the hepatic oxidative damage induced by Cd in rats (Al-Baqami and Hamza, 2021). The reversal effects of *Portulacae oleracea* against Cd-induced hepatorenal toxicities in mice have been reported (Tian et al., 2021).

As a regulator of cellular resistance to oxidants, Nrf-2 is an important defense factor against a variety of pathogenic processes, including oxidative damage, carcinogenesis, and numerous hazardous substances (Buha et al., 2021). In addition to controlling the adaptive response, Nrf-2 serves as a potent defense mechanism for living things against environmental toxins. After nuclear translocation, Nrf-2 negatively controls the inflammatory signaling pathway and inhibits oxidative stress by decreasing intracellular reactive oxygen species (ROS) levels (Saha et al., 2020). Nrf-2 acts as a transcriptional activator of antioxidants-responsive genes, the relationship between Nrf-2 and various liver injuries has been shown, and knowledge of the molecular processes that Nrf-2 modulates may help develop new therapeutic approaches for the management of liver illnesses (El-Said et al., 2023a). A previous study reported that natural products suppress heavy metals-induced toxicity via the cellular defense systems that were dependent on Nrf-2 (He et al., 2021).

*Urtica pilulifera* (stinging nettle) is classified as a popular plant that has been extensively cultivated in the Mediterranean region and belongs to the family *Urticaceae*. This annual herb has a straight, square-shaped leafy stem, a troublesome, branched rhizome, and all its components are covered in stinging hairs (Zamani and Razavi, 2021). Different parts of *Urtica* species have been reported for their potential phytochemical content including flavonoids and phenolic compounds that were linked to a variety of pharmacological and therapeutic applications (Taheri et al., 2022). The biomedical importance of *U. pilulifera* has been realized for the treatment of various diseases (El-Said et al., 2023b). Numerous *Urtica* species were commonly used to treat gout, fever, rheumatism, asthma, diabetes, diarrhea, eczema, hemorrhoids, scurvy, and tuberculosis. *Urtica* species have been primarily utilized to treat anemia, prostate enlargement, and as a diuretic. Furthermore, a previous report investigated the protective role of *U. dioica* against mercury-induced toxicity (Siouda and Abdennour, 2015). Therefore, this research investigated the possible ameliorative mechanism of *Urtica pilulifera* leaves extract (UPLE) against hepatotoxicity induced by cadmium in mice.

## 2 Materials and methods

### 2.1 Chemicals

Cadmium chloride (CdCl_2_, 99.99%) (Cat. no. 202908), potassium acetate, lead acetate, phenol, and sodium nitroprusside were purchased from Sigma Aldrich (Darmstadt, Germany). All chemicals and reagents used in this study were of high purity grades.

### 2.2 Collection and preparation of plant materials

The Crop Institute Agricultural Research Centre in Giza, Egypt is where the *U. pilulifera* leaves were collected. The plant met all applicable institutional requirements and was authenticated by a specialist. After being shade-dried, the leaves were pulverized into powder. A mixture of 50 g of leaves powder and 500 mL of 70% ethanol was filtered, then the *U. pilulifera* leaves extract (UPLE) was dried (Fakher El Deen et al., 2022).

### 2.3 Phytochemical analysis of UPLE

Total phenolic, flavonoid, total antioxidant capacity, saponin and anthocyanin were evaluated in UPLE (Hiai et al., 1975; Prieto et al., 1999; Singleton et al., 1999; Zhishen et al., 1999). A spectrophotometric assessment was used for DPPH radical scavenging capability (Blois, 1958). The chelation power of ferrous ions by UPLE was determined (Ebrahimzadeh et al., 2008).

### 2.4 Gas chromatography and mass spectrum (GC-MS) profiling of UPLE

Secondary metabolites of UPLE were detected using Trace GC 1310-ISQ mass spectrometer “GC-MS” (Thermo Scientific, Austin, TX, USA). By comparing the components’ retention times and mass spectra to those in the WILEY 09 and NIST 11 mass spectral databases, the components were identified.

### 2.5 Mice and experimental design

Forty male Swiss albino mice (25 ± 2 g, weight, and 7–8 weeks, age) were obtained from the National Research Center (NRC, Cairo, Egypt) The experimentation was performed in accordance with the moral standards established by Tanta University’s Faculty of Science’s Animal Care and Use Committee (ACUC-SCI-TU-088), Egypt. Four groups of ten mice each were used. The first (G1) was the negative control. G2 was injected i.p., with UPLE daily (100 mg/kg b.wt). G3 was injected i.p., with Cd daily (5 mg/kg b.wt) (El-Said et al., 2022). G4 was injected with Cd as in G3 and with UPLE as in G2. On day 11, % of body weight changes was calculated. Cd concentration was determined in liver tissues to screen its accumulation after UPLE treatment. To obtain blood for haematological evaluations, all mice were anesthetized using isoflurane, blood was collected from the dorsal pedal vein and sera were then separated for biochemical assessment. Moreover, liver tissues were prepared for biochemical, molecular, and histopathological investigations.

### 2.6 Hematological and biochemical analysis

Hematological parameters including RBC, Hb content, WBC, and platelets as well as the differential leucocytes were estimated using standard automated procedures. Measurement of aspartate aminotransferase (AST) (Cat. no. AS106145), alanine aminotransferase (ALT) (Cat. no. AL103145), alkaline phosphatase (ALP) (Cat. no. AP1020), total bilirubin (T.B) (Cat. no. BR 1111), gamma-glutamyl transferase (GGT) (Cat. no. GT 1471), and total proteins (Cat. no. TP 2020) were performed in serum by using their kit (Bio-diagnostic, Egypt). Hepatic malondialdehyde (MDA) (Cat. no. MD2529), superoxide dismutase (SOD) (Cat. no. SD2521), catalase (CAT) (Cat. no. CA2517), glutathione peroxidase (GPX) (Cat. no. GP2524), and glutathione reductase (GR) (Cat. no. GR2523) were determined by using their kit (Bio-diagnostic, Egypt) following the manufacturer protocols. IL-1ß and TNF-α were performed in liver homogenates using ELISA kits specified for mice according to the manufacturer protocols. IL-1ß (Cat. no. EM2IL1B), Thermo-Fisher Scientific, India Pvt. Ltd., and TNF-α (Cat. no. EZMTNFA), Millipore, Darmstadt, Germany. Furthermore, determination of hepatic nuclear Nrf-2 levels were evaluated using mouse Nrf-2 ELISA kit (catalog no. MBS2516218) from MyBioSource, Inc., San Diego CA, USA.

### 2.7 Real-time (RT-PCR) analysis

Using the GAPDH gene as an internal reference, the mRNA expression of SOD, CAT, GPX, GR, IL-1β, TNF-α, and Nrf-2 genes was assessed in liver tissues by SYBR Green. The primer pairs used were prepared using the Primer-Blast program from NCBI (Table 1).

**TABLE 1:** Forward and reverse primer sequences for real-time PCR.

Gene	Accession number	Forward sequence (5′–3′)	Reverse sequence (5′–3′)
SOD	NM_017050	CGA​GCA​TGG​GTT​CCA​TGT​C	CTG​GAC​CGC​CAT​GTT​TCT​TAG
CAT	NM_009804.2	CCGACCAGGGCATCAAAA	GAG​GCC​ATA​ATC​CGG​ATC​TTC
GPX	NM_001329527.1	CAG​CCG​GAA​AGA​AAG​CGA​TG	TTG​CCA​TTC​TGG​TGT​CCG​AA
GR	NM_010344.4	TGG​CAC​TTG​CGT​GAA​TGT​TG	CGA​ATG​TTG​CAT​AGC​CGT​GG
IL-1β	NM_008361.4	TGC​CAC​CTT​TTG​ACA​GTG​ATG	TTC​TTG​TGA​CCC​TGA​GCG​AC
TNF-α	NM_013693.3	AGA​GGC​ACT​CCC​CCA​AAA​GA	CGA​TCA​CCC​CGA​AGT​TCA​GT
Nrf-2	NM_010902.4	CCT​CTG​TCA​CCA​GCT​CAA​GG	TTC​TGG​GCG​GCG​ACT​TTA​TT
GAPDH	NM_001289726.1	TCA​CCA​CCA​TGG​AGA​AGG​C	GCT​AAG​CAG​TTG​GTG​GTG​CA

SOD, Superoxide dismutase; CAT, Catalase; GPX, Glutathione peroxides; GR, Glutathione reductase; IL-1β, Interleukin-1 beta; TNF-α, Tumor necrosis factor alpha; Nrf-2, Nuclear-related factor-2; GAPDH, Glyceraldehyde-3-phosphate dehydrogenase housekeeping gene.

### 2.8 Histopathological investigations

Following processing in various alcohol and xylene grades, the formalin-fixed liver sections were embedded in paraffin blocks. Hematoxylin and eosin-stained slices (5 μm) were inspected under an Optika light microscope (B-350) to examine hepatocytes (Bancroft and Gamble, 2008). The hepatic injuries were scored by examining histological indices, including inflammatory cell infiltration as well as alterations in hepatic cells, according to the methodology of de Menezes et al. (2019). The severity score for damages ranged from 0 to 4 (Figure 6).

### 2.9 Statistical analysis

The one-way ANOVA findings were analyzed using Graph Pad Prism software (San Diego, CA, USA); *p* < 0.05 was found to be statistically significant, and Tukey’s test was employed for multiple comparisons.

## 3 Results

### 3.1 Phytochemicals content of *Urtica pilulifera* leaves

The phytochemicals analysis of *Urtica pilulifera* leaves (UPL) showed promising phenolic and flavonoid contents (19.94 ± 1.25 mg GAE/g DW and 12.15 ± 3.15 mg QUE/g DW). The capacity of the total antioxidant recorded 256.28 ± 5.39 mg AE/g DW. Saponin and anthocyanin contents were 375.47 ± 4.76 mg*/*g DW and 3.28 ± 0.36 mg ECG/g DW, respectively (Table 2). The % of DPPH scavenging represented 78% ± 1.25, and its IC_50_ was 5.89 ± 0.45 mg/mL. Furthermore, the metal chelating activity of the UPL showed potential chelation power against metals *in vitro* that represented 84% ± 3.65 with an EC_50_ of 375.72 ± 4.41 μg/mL (Table 2).

**TABLE 2:** Phytochemical analysis of *U. pilulifera* leaves (UPL).

Phytochemical analysis	UPL
Total phenolic (mg GAE/g DW)	19.94 ± 1.25
Total flavonoids (mg QE/g DW)	12.15 ± 3.15
TAC (mg AAE/g DW)	0.48 ± 0.07
Saponin (mg/g DW)	375.47 ± 4.76
Anthocyanin (mg ECG/g DW)	3.28 ± 0.36
DPPH scavenging (%)	78% ± 1.25
IC_50_ of DPPH (mg/mL)	5.89 ± 0.45
Metal chelating activity (MCA) (%)	84% ± 3.65
EC_50_ of MCA (μg/mL)	375.72 ± 4.41

DW, Dry weight; GAE, Gallic acid equivalent; QUE, Quercetin equivalent. TAC, Total antioxidant capacity; ECG, Epicatechin gallate; AAE, Ascorbic acid equivalent; DPPH, 2,2-diphenyl-1-picrylhydrazyl; IC_50_, Half maximal inhibitory concentration; EC_50_, Half maximal effective concentration.

### 3.2 GC-MS analysis of *Urtica pilulifera* leaves extract

**FIGURE 1:**
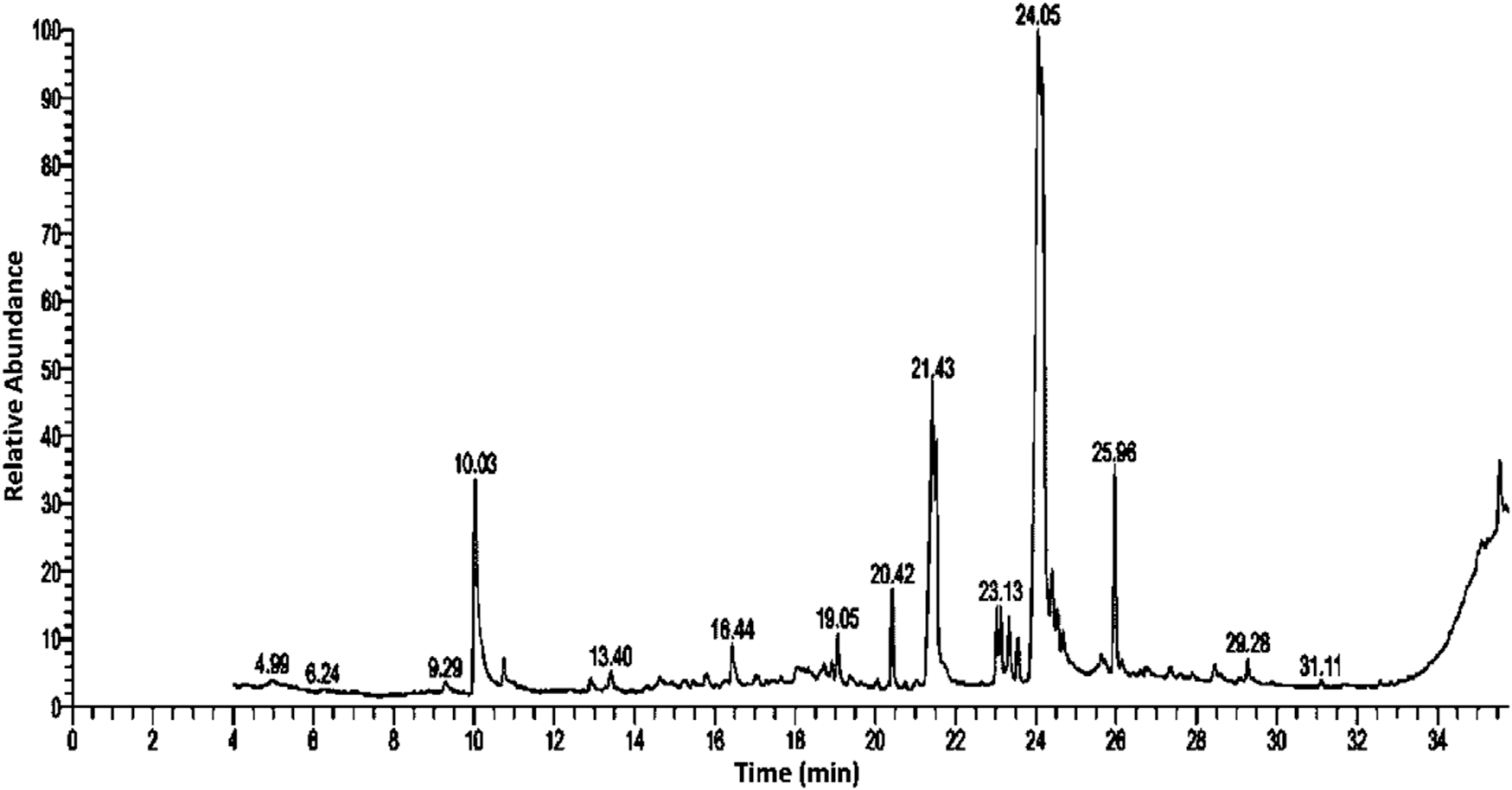
GC-MS profile chromatogram of *Urtica pilulifera* leaves extract.

**FIGURE 2:**
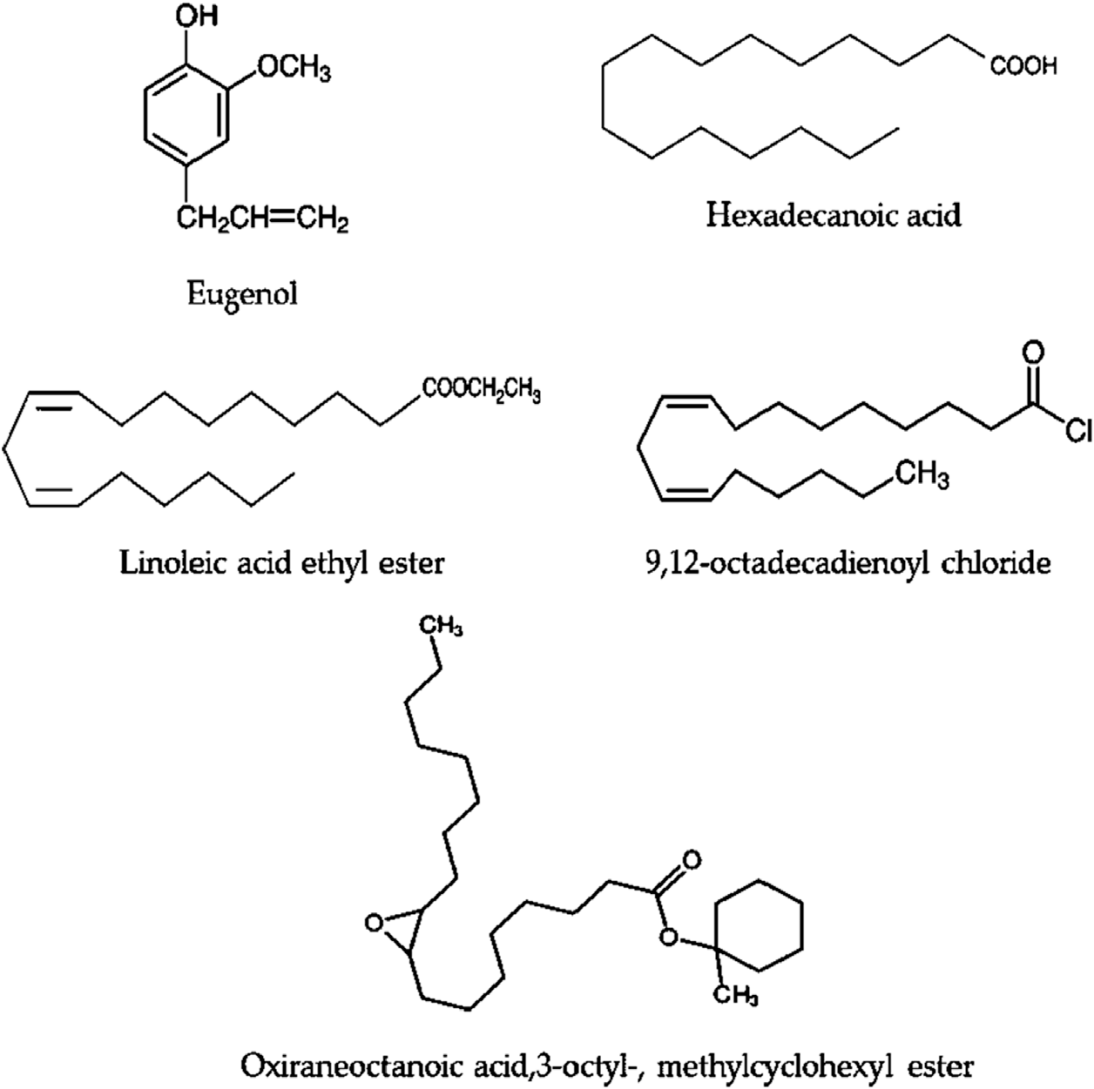
The phytochemical components that are most prevalent in *Urtica pilulifera* leaves extract.

Bioactive secondary metabolites present in UPLE were detected using GC-MS technique. The results showed that the most abundant chemical compounds detected in UPLE were eugenol, hexadecanoic acid, linoleic acid ethyl ester, 9,12-octadecadienoyl chloride, and Oxiraneoctanoic acid,3-octyl, methylcyclohexyl ester (Figures 1, 2; Table 3). These phytochemicals were recorded at the retention times (RT) 10.02, 21.44, 24.05, 24.18, and 25.96, respectively. The peak areas percentages (PA%) were 9.27%, 11.40%, 22.15%, 22.82%, and 7.18%, respectively.

**TABLE 3:** GC-MS analyses of *Urtica pilulifera leaves* extract (UPLE).

No.	RT (min.)	Name	M. F.	P.A %
1	10.02	Eugenol	C_10_H_12_O_2_	9.27
2	13.52	1,1-Bicyclopropyl-2-octanoic acid 2hexyl-methyl ester	C_21_H_38_O_2_	3.69
3	16.44	Trans-13-Octadecenoic acid	C_18_H_34_O_2_	1.41
4	19.05	2-Pentadecanone, 6,10,14-trimethyl	C_18_H_36_O	1.68
5	20.42	Hexadecanoic acid, methyl ester	C_17_H_34_O_2_	3.14
6	21.44	Hexadecanoic acid	C_16_H_32_O_2_	11.40
7	21.58	Hexadecanoic acid, ethyl ester	C_18_H_36_O_2_	5.57
8	23.03	9,12-Octadecadienoic acid, methyl	C_19_H_34_O_2_	2.28
9	23.12	11-Octadecenoic acid, methyl ester	C_19_H_36_O_2_	2.23
10	23.33	Phytol	C_20_H_40_O	2.15
11	23.55	Methyl stearate	C_19_H_38_O_2_	1.39
12	24.05	Linoleic acid ethyl ester	C_20_H_36_O_2_	22.15
13	24.18	9,12-Octadecadienoyl chloride	C_18_H_31_ClO	22.82
14	24.41	9-Octadecenoic acid	C_18_H_34_O_2_	2.07
15	24.54	Octadecanoic acid, ethyl ester	C_20_H_40_O_2_	1.29
16	25.96	Oxiraneoctanoic acid,3-octyl-, methylcyclohexyl ester	C_19_H_36_O_3_	7.18
17	32.55	α-Sitosterol	C_29_H_50_O	2.48

RT, Retention time; M.F., Molecular formula; P.A%, Peak area percentage.

### 3.3 Effect of UPLE treatment on the percentages of body weight changes

**FIGURE 3:**
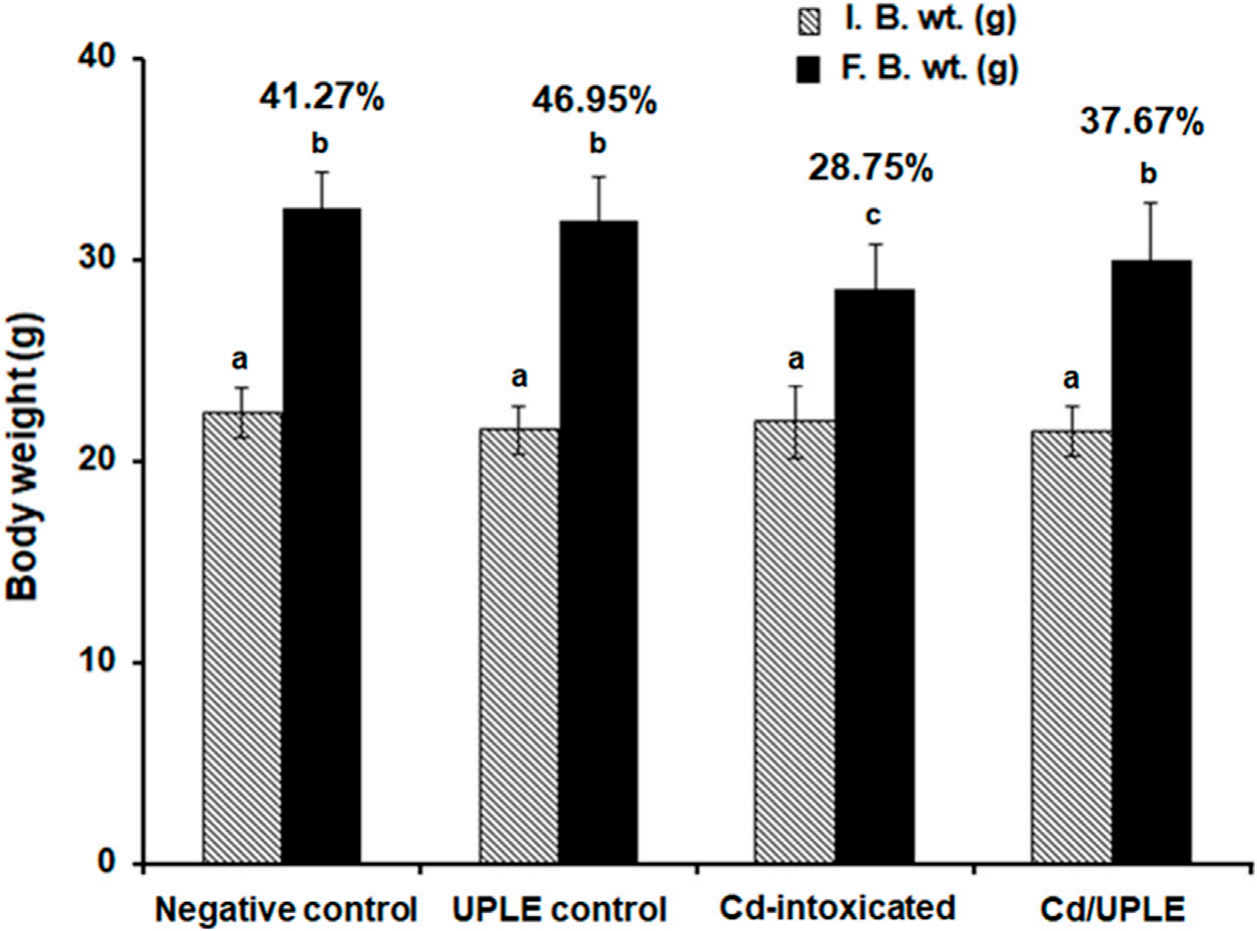
Effect of UPLE on the percentages of body weight changes of cadmium-intoxicated mice. I. B. wt.: Initial body weight; F. B. wt.: Final body weight; UPLE: *Urtica pilulifera leaves* extract; Cd: Cadmium. The values represented as means ± S.D (*n* = 10). Means that do not share a letter in each column are significantly different (*p* ≤ 0.05).

The % b.wt changes of the negative control and UPLE-administered groups were 41.27% ± 2.56% and 46.59% ± 3.25, respectively. The group that was injected with Cd showed a significant decrease in the % b. wt change to 28.75% ± 2.74 (*p* ≤ 0.05) (Figure 3). However, the treatment of Cd-intoxicated mice with UPLE led to improvement in body weight by significant increase in the % of b. wt change to 37.67 ± 2.43 (*p* ≤ 0.05) (Figure 3).

### 3.4 Effect of UPLE treatment on Cd concentration in the liver of mice treated with CdCl_2_

**FIGURE 4:**
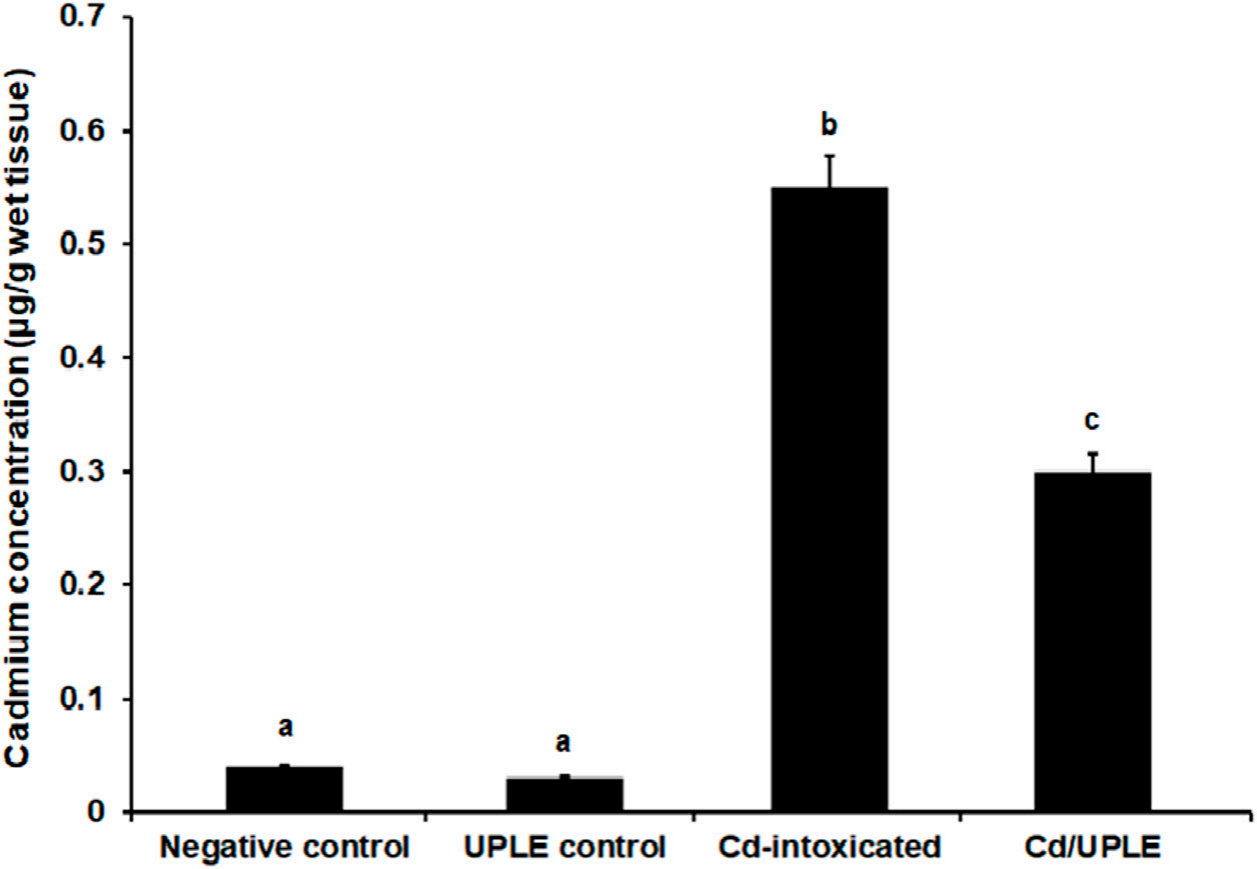
Effect of UPLE on cadmium concentration in liver tissues of cadmium-intoxicated mice. UPLE: *Urtica pilulifera leaves* extract; Cd: Cadmium. The values represented as means ± S.D (*n* = 10). Means that do not share a letter in each column are significantly different (*p* ≤ 0.05).

Compared to the control groups, the group treated with CdCl_2_ alone showed higher concentrations of Cd in the liver tissue (0.56 ± 0.08 μg/g wet tissue) (Figure 4). Treatment with UPLE affected Cd concentrations in liver of mice treated with CdCl_2_ by significantly decreasing (*p* ≤ 0.05) Cd concentration compared to those in the Cd-intoxicated mice without treatment.

### 3.5 Effect of UPLE treatment on hematological parameters

The results showed that there were no significant changes in the hematological parameters among the negative control and UPLE-administrated groups. However, the Cd-injected group showed significant decrease (*p* < 0.05) in the RBCs count to 6.37 ± 0.85 × 10^6^/µL and in Hb level to 9.78 ± 0.67 g/dL versus their control. The group of mice which treated with Cd/UPLE showed significant restoration of RBCs count and Hb concentrations (Table 4).

**TABLE 4:** Effect of UPLE on the hematological parameters in Cd-intoxicated mice.

Groups	RBCs (x10^6^/µL)	Hb (g/dL)	WBCs (x10^3^/µL)	Platelets (x10^3^/µL)
Negative control	9.65 ± 1.52^a^	12.95 ± 1.13^a^	8.19 ± 0.55^a^	882.51 ± 51.54^a^
UPLE control	9.81 ± 1.27^a^	13.11 ± 1.07^a^	7.92 ± 0.85^a^	890.76 ± 76.27^a^
Cd-intoxicated	6.37 ± 0.85^b^	9.78 ± 0.67^b^	11.68 ± 0.37^b^	1,057.15 ± 65.33^b^
Cd/UPLE	8.75 ± 0.92^c^	11.54 ± 0.57^a^	9.27 ± 0.59^a^	945.37 ± 70.41^c^

The values represent mean ± S.D. (n = 10). UPLE, *Urtica pilulifera leaves* extract; Cd, Cadmium; RBCs, Red blood cells; Hb, Hemoglobin; WBCs, White blood cells. Means that do not share a letter in each column were significantly different (*p* < 0.05).

The total counts of WBCs and platelets were significantly increased (*p* < 0.05) in the group of mice injected with Cd alone (11.68 ± 0.37 × 10^3^/µL and 1,057.15 ± 65.33 × 10^3^/µL, respectively) when compared to negative control (8.19 ± 0.55 × 10^3^/µL and 882.51 ± 51.54 × 10^3^/µL, respectively). Treating Cd-injected mice with UPLE led to significant decrease (*p* < 0.05) in WBCs and platelet counts (9.27 ± 0.59 × 10^3^/µL and 945.37 ± 70.41 × 10^3^/µL, respectively) when compared to mice injected with Cd alone (Table 4). Furthermore, the injection of mice with Cd led to an increase in the percentages of neutrophils (%) and monocytes (%), while decreasing the percentage of lymphocytes (%). Treatment with UPLE with Cd injection led to modulation of the percentage of the differential leucocytes (Table 5).

**TABLE 5:** Effect of UPLE on the differential leukocytes in Cd-intoxicated mice.

Groups	Neutrophiles (x10^3^/µL)	Lymphocytes (x10^3^/µL)	Monocytes (x10^3^/µL)
Negative control	1.51 ± 0.09^a^	6.50 ± 1.1^a^	0.18 ± 0.03^a^
UPLE control	1.58 ± 0.07^a^	6.12 ± 1.5^a^	0.22 ± 0.05^a^
Cd-intoxicated	5.52 ± 0.10^b^	5.50 ± 1.4^b^	0.66 ± 0.06^b^
Cd/UPLE	2.88 ± 0.15^c^	5.98 ± 1.3^a^	0.41 ± 0.04^c^

The values represent mean ± S.D. (*n* = 10). UPLE, *Urtica pilulifera leaves* extract; Cd, Cadmium. Means that do not share a letter in each column were significantly different (*p* < 0.05).

### 3.6 Effect of UPLE treatment on biochemical parameters

Treatment of naïve mice with UPLE did not influence liver enzyme (AST and ALT), ALP, T.B, GGT, and total protein levels. However, mice that were intoxicated with CdCl_2_ showed significant increases in AST, ALT, ALP, T.B, and GGT levels and significant decrease in the total proteins level (*p* < 0.05) compared to the normal control. The Cd-injected mice with UPLE treatment restored the previously mentioned parameters close to the control group (Table 6 **)**.

**TABLE 6:** Effect of UPLE on the biochemical parameters in Cd-intoxicated mice.

Groups	AST (U/L)	ALT (U/L)	ALP (U/L)	T.B. (mg/dL)	GGT (U/L)	T. protein (g/dL)
Negative control	36.3 ± 1.23^a^	24.2 ± 0.93^a^	136.7 ± 5.15^a^	0.35 ± 0.012^a^	14.4 ± 0.28^a^	6.5 ± 0.19^a^
UPLE control	32.8 ± 1.12^a^	21.8 ± 0.71^a^	129.6 ± 4.21^a^	0.37 ± 0.014^a^	12.1 ± 0.37^a^	6.84 ± 0.15^a^
Cd-intoxicated	83.7 ± 3.95^b^	59.7 ± 1.76^b^	285.7 ± 7.52^b^	0.69 ± 0.015^b^	41.6 ± 0.96^b^	3.9 ± 0.17^b^
Cd/UPLE	49.9 ± 2.78^c^	32.3 ± 1.02^c^	195.8 ± 9.71^c^	0.43 ± 0.013^c^	22.3 ± 0.65^c^	5.3 ± 0.13^a^

The values represent mean ± S.D. (*n* = 10). UPLE, *Urtica pilulifera leaves* extract; Cd, Cadmium; AST, Aspartate transaminase; ALT, Alanine transaminase; ALP, Alkaline phosphatase; T.B., Total bilirubin; GGT, Gamma-glutamyl transpeptidase. Means that do not share a letter in each column were significantly different (*p* < 0.05).

When compared to the control groups, the Cd-intoxicated group had a 2.2-fold rise in hepatic MDA levels. Mice that were treated with Cd/UPLE showed a significant decrease (*p* ≤ 0.05) in the MDA level compared to the Cd-injected group (33.69 ± 1.02 nmol/g tissue versus 49.79 ± 1.37 nmol/g tissue) (Table 7). In contrast, the hepatic enzymatic antioxidants (SOD, CAT, GPX, and GR) were significantly reduced (*p* ≤ 0.05) in the Cd-intoxicated group. However, in comparison to the Cd-intoxicated group, the treatment with UPLE resulted in a significant improvement in the previous hepatic antioxidants activity (Table 7).

**TABLE 7:** Effect of UPLE on the oxidants/antioxidant’s status in Cd-intoxicated mice.

Groups	MDA (nmol/g tissue)	SOD (U/mg protein)	CAT (U/mg protein)	GPX (U/mg protein)	GR (U/mg protein)
Negative control	23.5 ± 0.92^a^	8.3 ± 0.33^a^	74.3 ± 2.41^a^	1.8 ± 0.04^a^	18.35 ± 0.45^a^
UPLE control	25.2 ± 1.55^a^	9.6 ± 0.28^a^	79.9 ± 3.52^a^	2.1 ± 0.09^a^	20.49 ± 0.80^a^
Cd-intoxicated	49.7 ± 1.37^b^	5.7 ± 0.09^b^	48.6 ± 1.15^b^	0.9 ± 0.06^b^	7.58 ± 0.25^b^
Cd/UPLE	33.9 ± 1.02^e,c^	7.6 ± 0.17^a^	69.5 ± 1.59^a,e^	1.4 ± 0.05^a,b^	13.94 ± 0.37^c^

The values represent mean ± S.D. (*n* = 10). UPLE, *Urtica pilulifera leaves* extract; Cd, Cadmium; MDA, Malondialdehyde; SOD, Superoxide dismutase; CAT, Catalase; GPX, Glutathione peroxides; GR, Glutathione reductase. Means that do not share a letter in each column were significantly different (*p* ≤ 0.05).

### 3.7 Cd/UPLE treatment mitigated inflammation induced by Cd and promoted Nfr2 signaling

**FIGURE 5:**
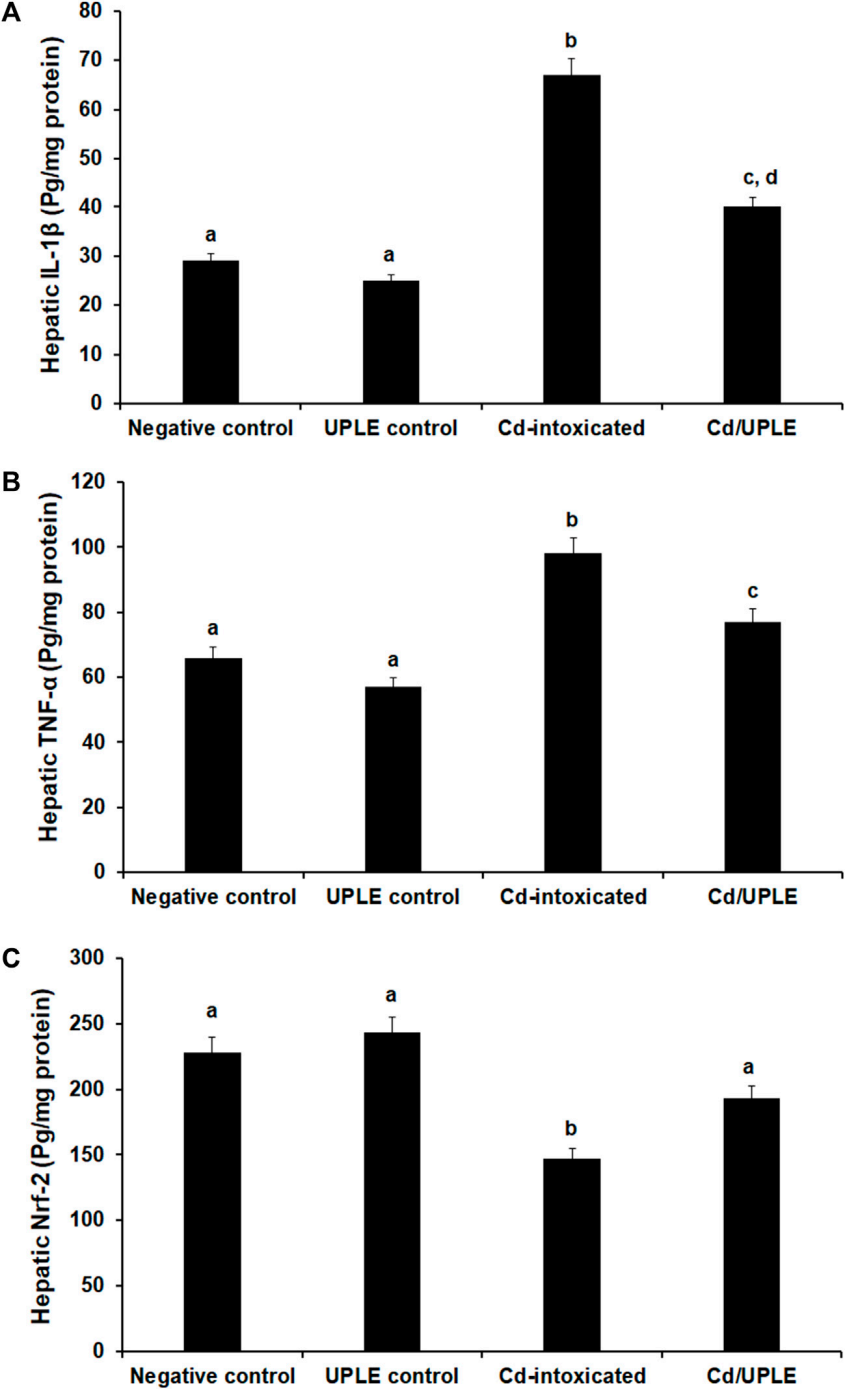
Effect of UPLE on hepatic inflammatory biomarkers and nuclear factor erythroid 2-related factor 2 of cadmium-intoxicated mice. **(A)** Interleukin-1β (IL-1β), **(B)** Tumor necrosis factor-α (TNF-α), and **(C)** Nuclear factor erythroid 2-related factor 2 (Nrf-2). UPLE: *Urtica pilulifera leaves* extract; Cd: Cadmium The values represent means ± S.D. (*n* = 10). Means that do not share a letter were significantly different (*p* < 0.05).

The results showed that there was a substantial increase (*p* < 0.05) in the IL-1β and TNF-α levels of the Cd-intoxicated group compared to that in the control groups; however, the treatment of Cd-injected mice with UPLE prompted a significant restoration in the levels of those markers (Figures 5A, B). Moreover, the group received a Cd intraperitoneally showed a significant decrease (*p* < 0.05) in hepatic Nfr-2 to 147.56 ± 2.85 pg/mg protein versus the normal control (228.47 ± 3.45 pg/mg protein) and UPLE control groups (243.78 ± 2.95 pg/mg protein). As compared to Cd-intoxicated mice, UPLE significantly increased Nfr-2 to 193.97 ± 3.05 pg/mg protein (Figure 5C).

### 3.8 Effect of Cd/UPLE treatment on molecular analysis

The results of the qRT-PCR approach were consistence with the ELISA method, indicating that within the group that had been exposed to Cd, there was significant downregulation (*p* < 0.001) in the mRNA expression levels of the antioxidant genes (SOD, CAT, GPX, and GR) accompanied by a marked upregulation in the expression levels of IL-1β and TNF-α, when compared to control groups. Treatment of Cd-administered mice with UPLE led to significant modulation of the gene expression of the previous genes close to control groups (Tables 8, 9). The results revealed that post Cd intoxication, the Nrf-2 mRNA expression in hepatic tissues was significantly downregulated (*p* < 0.001). However, UPLE treatment significantly upregulated Nrf-2 expression in the Cd-injected mice (Tables 8, 9).

**TABLE 8:** Effect of UPLE on the hepatic antioxidant genes expression in Cd-intoxicated mice.

Groups	SOD	CAT	GPX	GR
Negative control	1.07 ± 0.03^a^	1.08 ± 0.04^a^	1.00 ± 0.01^a^	1.01 ± 0.02^a^
UPLE control	1.65 ± 0.06^a^	1.95 ± 0.09^b^	2.05 ± 0.10^b^	1.54 ± 0.08^a^
Cd-intoxicated	0.30 ± 0.08^b^	0.56 ± 0.05^c^	0.48 ± 0.08^c^	0.35 ± 0.06^b^
Cd/UPLE	0.76 ± 0.05^c^	0.90 ± 0.08^a^	0.81 ± 0.07^a^	0.74 ± 0.09^a^

The values represent mean ± S.D. (*n* = 10). UPLE, *Urtica pilulifera leaves* extract; Cd, Cadmium; SOD, Superoxide dismutase; CAT, Catalase; GPX, Glutathione peroxidase; GR, Glutathione reductase. Means that do not share a letter in each column were significantly different (*p* < 0.001).

**TABLE 9:** Effect of UPLE on the hepatic inflammatory and nuclear-related factor-2 genes expression in Cd-intoxicated mice.

Groups	IL-1β	TNF-α	Nrf-2
Negative control	1.01 ± 0.03^a^	1.00 ± 0.04^a^	1.00 ± 0.01^a^
UPLE control	1.02 ± 0.02^a^	1.05 ± 0.06^a^	1.82 ± 0.03^b^
Cd-intoxicated	4.37 ± 0.16^b^	3.95 ± 0.19^b^	0.45 ± 0.06^c^
Cd/UPLE	2.95 ± 0.18^c^	2.38 ± 0.15^c^	0.91 ± 0.05^d^

The values represent mean ± S.D. (*n* = 10). UPLE, *Urtica pilulifera leaves* extract; Cd, Cadmium; IL-1β, Interleukin-1 beta; TNF-α, Tumor necrosis factor alpha; Nrf-2, Nuclear-related factor-2. Means that do not share a letter in each column were significantly different (*p* < 0.001).

### 3.9 Treatment with UPLE restored hepatic histopathological changes induced by Cd

**FIGURE 6:**
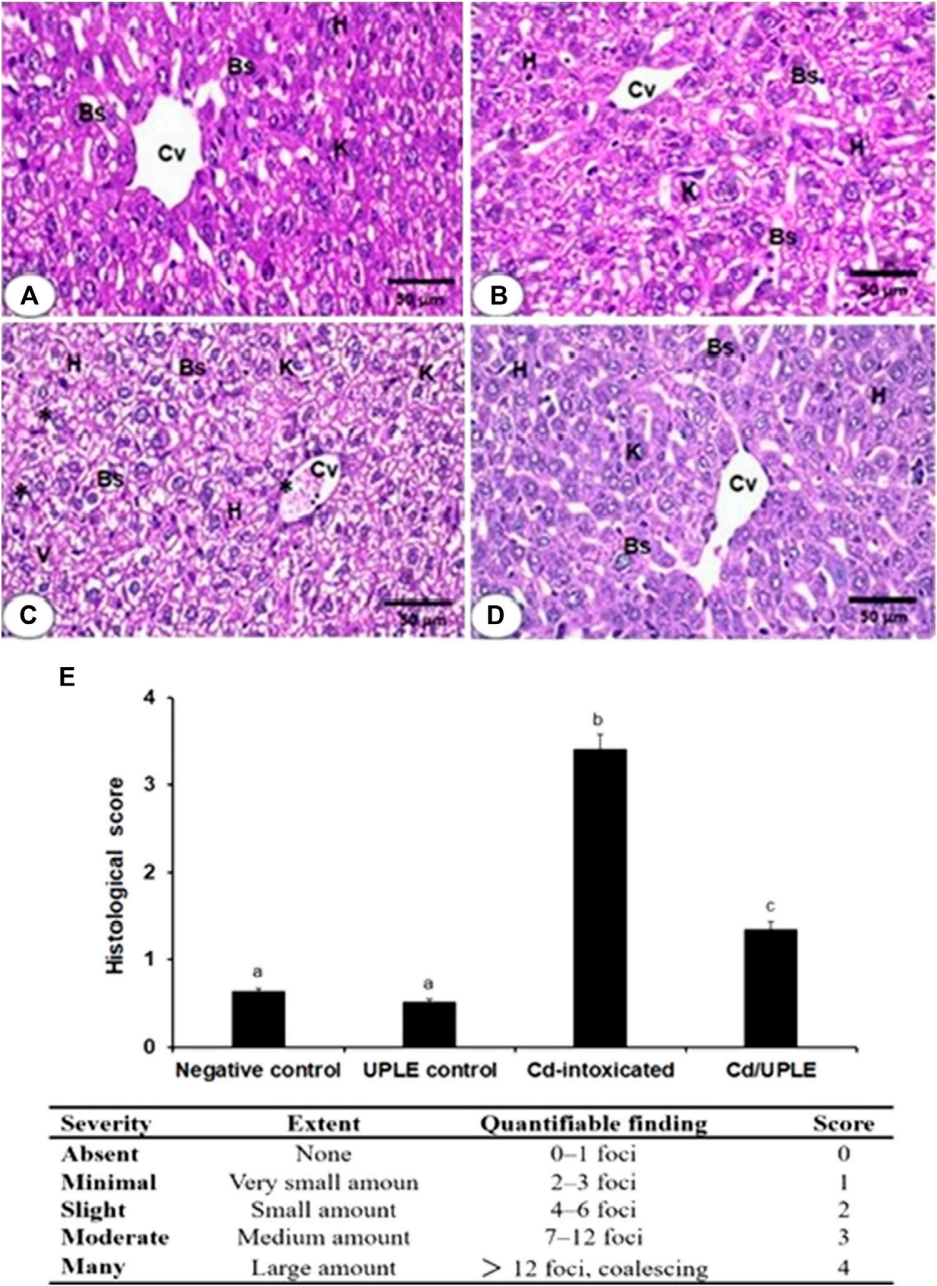
**(A)** The liver section photomicrograph of the normal control group demonstrates the typical hepatic architectures, including regular central veins (CV), normal hepatocytes (H), normal blood sinusoids (Bs), and Kupffer cells (K) **(B)** The majority of the hepatocytes in the UPLE control group’s liver are normal, with normal Bs and Ks **(C)** The Cd-injected group’s liver section shows abnormal blood sinusoids with distinct K, cellular infiltrations (*), vacuolated and degraded cytoplasm (V), and disorganized hepatic architecture **(D)** The liver section of the Cd/UPLE treated group exhibits improved hepatic organization with reduced cellular infiltrations and congestion. (X 400). **(E)** Histopathological scoring displaying infiltration of inflammatory cells and hepatic damages in Cd-intoxicated group. The values represent means ± S.D. (*n* = 10). Means that do not share a letter were significantly different (*p* < 0.05).

Liver sections of the negative control and UPLE control groups demonstrated normal hepatocyte morphology (Figures 6A, B). In liver section of the CdCl_2_ group was characterized by hepatocyte disarrangement, cellular degeneration, and portal vein congestion (Figure 6C). On the other hand, Cd-induced severe hepatic lesions in mice with Cd injury treated with UPLE were significantly reduced (Figure 6D). Histological scoring displayed significant (*p* < 0.05) injuries in the hepatic tissues of Cd-injected mice (Figure 6E). However, treatment of Cd-injected mice with UPLE significantly (*p* < 0.05) alleviated the detrimental alterations in liver architecture compared with the Cd group (Figure 6E).

## 4 Discussion

Over the past few decades, there has been a surge in heavy metal contamination in the environment, which poses major hazards to all biological systems. Cadmium (Cd) is an extremely toxic heavy metal that is transferred to humans, where it damages vital organs including liver, it is a cumulative non-biodegradable element; its biological half-life is 10–30 years (Khafaga et al., 2019). Cd could bind to sulfhydryl groups on critical molecules including enzymes in liver cells causing hepatic dysfunction. Cadmium toxicity cannot be specifically treated in a way that is safe and effective, therefore, researchers have sought an effective Cd poisoning remedy from natural sources paying close attention to how antioxidants generated from plants protect. Natural products have been suggested to stop the harm caused by exposure to Cd (Khafaga et al., 2019). *Urtica pilulifera* showed powerful medicinal importance due to its promising phytoconstituents. A broad spectrum of pharmacological activities of *Urtica* have been reported including antioxidant, anticancer, antimicrobial, and anti-inflammatory effects (Taheri et al., 2022). A previous study reported significant antioxidant and hepatoprotective potentials of *Urtica* (Joshi et al., 2015). Therefore, this study evaluated the bioactive constituents of *Urtica pilulifera* leaves and their role in counteracting the hepatotoxic effects induced by Cd in mice.

The current study showed that UPLE had adequate quantities of phytochemical constituents that agreed with the previous report, which demonstrated various phytochemicals in *Urtica* including flavonoids and phenolic chemicals, the pharmacological and therapeutic effects of which have been extensively documented (Taheri et al., 2022). The DPPH scavenging activity of UPL in this study was 78%, and its IC_50_ was 5.89 ± 0.45 mg/mL. These results were in accordance with a previous study that screened the antioxidant activity of *Urtica* and reported a DPPH scavenging activity of 77.85% with an IC_50_ of 4.69 mg/mL (Belmamoun et al., 2023). Moreover, the chelating activity of hydroalcoholic extracts of UPL in the present study showed a percentage chelation of 84%, and the EC_50_ value was 375.72 μg/mL. These findings were in line with the previous study by Ozen et al. (2010) who reported the antioxidant properties of *U. pilulifera* leaves extract. In the current study, GC-MS analysis of UPLE showed the presence of potent phytochemicals. A previous report screened the phytochemicals of *Urtica dioica* leaves by GC-MS methods. They indicated the presence of bioactive chemical constitutions, which could be useful for various herbal formulations as anti-inflammatory (Al-Tameme et al., 2015). Interestingly, eugenol, hexadecanoic acid, linoleic acid ethyl ester, 9,12-octadecadienoyl chloride, and Oxiraneoctanoic acid, 3-octyl, methylcyclohexyl ester were mainly detected in UPLE, which supply a variety of biological functions. Eugenol is a phenolic aromatic compound known for its potential antibacterial, antiviral, antifungal, antioxidant, anti-inflammatory, and anticancer properties. It has been utilized for a long time in several pharmacological and medical fields (Ulanowska and Olas, 2021). The anti-inflammatory property of hexadecanoic acid has been reported for the treatment of rheumatic symptoms. Also, linoleic acid esters showed potential anti-inflammatory activity in mammalian cells (Kolar et al., 2019). Furthermore, 9,12-octadecadienoyl chloride is a bioactive compound that has been reported for its strong antioxidant properties (Khan et al., 2021).

This study revealed a significant decrease in the % BW of the Cd-intoxicated group; this could result from the harmful impacts of Cd heavy metal on the vital tissues and the dysfunction in the glucocorticoid system in Cd-intoxicated mice. Moreover, the impairment of glucocorticoid hormones, which were involved in the metabolism of fats, proteins, and glucose, may provide an explanation. This agreed with previous study that reported the impact of Cd intoxication on the BW of experimental animals (El-Said et al., 2022). Treatment of Cd-injected mice with UPLE produced a notable improvement in the percentage BW, this could be due to the elimination of the Cd toxic effects from the mice’s circulation and tissues, improving nutrition status and metabolism, which suggests the ameliorative effect of UPLE on Cd toxicity. This result was consistent with earlier studies showing the impact of pharmaceuticals on the enhancement of body weight loss brought about by a cadmium injection (El-Said et al., 2022). When cadmium enters the body, it mixes with metallothionein, which leads to liver accumulation, which is responsible for the metabolism of toxic chemicals (Ramakrishnan et al., 2017). In this study, the treatment of Cd-intoxicated mice with UPLE significantly decreased the concentration of Cd in liver tissues. This could indicate the chelating properties and therapeutic effects of UPLE against liver injury promoted by Cd. This data was in accordance with previous studies that reported the potential role of *Urtica* in mitigating liver damage and the effect of natural products on decreasing Cd accumulation in liver tissues (Joshi et al., 2015; Siouda and Abdennour, 2015; Almeer et al., 2018).

It has been reported that Cd injection into mice increased myeloid and monocytic cells in bone marrow (Kacar Kocak, et al., 2010). In this investigation, the outcomes revealed that Cd injection in mice caused significant alterations in the hematological parameters, including RBCs, Hb, WBCs, and platelets. Cd injection, furthermore, increased the percentages of neutrophils (%) and monocytes (%), while decreasing the percentage of lymphocytes (%). These alterations could be the result of Cd-induced oxidative damage to the erythrocytes, which increased lipid peroxidation, oxidative stress, the degradation of the cell membrane, and the occurrence of systemic inflammation. These results agreed with a previous study that demonstrated that Cd injection caused anemia, thrombocytosis, and a decrease in lymphocytes in experimental animals (Andjelkovic et al., 2019). The neutrophilia with leukocytosis observed in the current study after Cd injection could be due to the release and mobilization of neutrophils (Kataranovski et al., 2009). Treatment with UPLE improved the alterations in the hematological parameters that were induced by Cd injection in mice, as evidenced by restoration of RBCs, WBCs, and platelets counts. This data suggested the improvement of bone marrow thrombopoietin and megakaryocytopoiesis by UPLE treatment. Our results agreed with previous studies reporting the effects of plants extracts on the improvement of hematological indices after cadmium intoxication in experimental animals by increasing erythropoietin and scavenging free radical-induced damage in cells (Andjelkovic et al., 2019; Oyewopo et al., 2020; El-Said et al., 2022; Sani et al., 2023).

Our results revealed that administration with CdCl_2_ for 10 days exerted hepatic disability in mice which was confirmed by the significant increase of serum AST, ALT, ALP, T.B, and GGT levels and a significant decrease in the total protein level due to hepatocyte leakage and impaired liver synthetic functions. Possible causes of elevated bilirubin include the hemolysis of erythrocytes and other hemoglobin-containing proteins that were confirmed in hematological analysis. Another explanation could be that exposure to Cd is known to cause the production of ROS, which can cause oxidative damage to tissues and organs. The administration of UPLE attenuated Cd-induced hepatotoxicity, as shown by the decreased levels of hepatic markers, which clearly indicates that UPLE stabilizes the cell membrane in hepatic damage induced by Cd. The stabilization of biliary dysfunction in mice’s liver during hepatic injury is indicated by the simultaneous suppression of elevated bilirubin levels and the depletion of enhanced ALP activity. The successful management of ALP and bilirubin levels in the treated groups suggests that the hepatocyte secretary mechanism has improved. It has been reported that *Urtica* decreases the liver marker enzymes in CCl_4_-challenged rats (Joshi et al., 2015).

Because of an imbalance in the antioxidant/oxidant status, which causes the oxidation of biological components, Cd exerts harmful effects through structural, functional impairment in liver cells, ROS generation, oxidative stress-neutralizing enzymes disruptions, and then acute hepatotoxicity. This hepatotoxicity is caused by the destructive effects of free radicals which can be potentially reversed by plants-derived antioxidants (Almeer et al., 2018). Elevated MDA levels, as demonstrated in experimental mice administered with Cd, indicate increased lipid peroxidation resulting in tissue damage and the breakdown of antioxidant defense mechanisms. The primary cause of hepatotoxicity, lipid peroxidative degradation of the bio-membrane, also has an impact on the liver’s antioxidant system. Furthermore, Cd can lower the amounts of SOD, GSH, GSH-Px, and CAT in soft tissues because of its strong affinity for sulfhydryl groups (Elkhadragy and Abdel Moneim, 2017).

The current study reported that the treatment of Cd-injected mice with UPLE led to a marked inhibition of lipid peroxidation along with restoration of the antioxidants’ status at the biochemical and molecular levels. This could be one possible mechanism to combat hepatic oxidative stress induced by cadmium intoxication. UPLE prevents hepatic tissue damage, thereby maintaining membrane integrity, which in turn maintains the level of antioxidants. These data were in tandem with previous studies addressed the impacts of plants extracts on Cd-induced oxidative stress and hepatotoxicity in experimental animals (Almeer et al., 2018; Unsal et al., 2020; Al-Baqami and Hamza, 2021; Mobasher et al., 2021; El-Said et al., 2022).

Numerous inflammatory processes triggered by oxidative stress through cascade of cellular signals. The Kupffer cells activation and induced inflammatory mediators in the liver tissues contribute to free radicals’ generation. Notably, we confirmed in this study that exposure to cadmium can also drive an inflammatory response in liver, which led to a profound increase in the level of IL-1β and TNF-α in mice hepatocytes.

The overproduction of these inflammatory markers may be due to the immune-modulatory action of Cd on immune cells. These results are supported by the findings of previous researchers, who have reported rises in the levels of TNF-α and IL-6 (Bhattacharjee et al., 2020). Treatment with UPLE reduced Cd toxicity by significantly ameliorating the inflammation, which suggests the anti-inflammatory properties of UPLE. Previous studies reported that plants extraction improves Cd-hepatotoxicity by remarkably reduction of inflammatory biomarkers (Noor et al., 2022; Nofal et al., 2023). A previous study encouraged the traditional use of *Urtica pilulifera* extract as an antioxidant and anti-inflammatory agent (Abo-elmatty et al., 2013). Furthermore, *Urtica* was reported to attenuate ovalbumin-induced inflammation in rats (Zemmouri et al., 2017).

Redox-constrained one essential transcription factor that regulates cellular antioxidant defenses was Nrf-2, which is potentially important against heavy metals-induced toxicity (Buha et al., 2021). In this investigation, the intraperitoneal injection of Cd in mice resulted in a remarkable reduction in hepatic Nfr-2. A previous study reported that cadmium induces acute liver injury by inhibiting the Nrf-2 signaling pathway in mice (Liu et al., 2020). Interestingly, UPLE lessened the toxicity of Cd on liver by increasing Nrf-2 expression. Thereby enhancing the antioxidant defense system to prevent Cd-induced oxidative damage. Due to Nrf-2’s high sensitivity to Cd, Cd-induced apoptosis is prevented when it is overexpressed. These data were in harmony with previous studies that reported the Nrf-2 signaling pathway as a promising therapeutic target by natural products in cadmium toxicity and initiated the transcription of genes encoding downstream antioxidant enzymes (He et al., 2021; Wang et al., 2022).

One well-known hazardous element that may have caused some hepatocyte damage is cadmium. In this investigation, histo-morphological assessment of hepatic tissues indicated that the mice’s exposure to Cd resulted in detrimental liver histopathological aberrations. Our findings coincide with previous reports, in which it was revealed that the Cd caused histological alterations in hepatic tissues of experimental animals (Gattea Al-Rikabi et al., 2021; Noor et al., 2022). Liver architectures were well preserved with less congestion and fewer cellular infiltrations upon the treatment of Cd-injured mice with UPLE, which suggests the histo-protective effect of UPLE treatment against Cd intoxication. The advantageous effects of UPLE, which contains a variety of bioactive compounds with well-established documented the therapeutic and antioxidant characteristics, may be responsible for the reported outcomes due to the chelation of Cd and stopping its harmful effects on the hepatocytes. These findings were in accordance with several previous studies that reported the impact of natural products on Cd-induced histopathological liver alterations (Almeer et al., 2018; Al-Baqami and Hamza, 2021; El-Said et al., 2022; Noor et al., 2022).

Collectively, UPLE reduced Cd-induced liver dysfunctions through its antioxidative and anti-inflammatory properties by enhancing Nrf-2 signaling and antioxidant enzyme gene expression in the liver of mice. Interestingly, this study is the first to explore the beneficial role of UPLE treatment in Cd-induced liver damage. Therefore, UPLE could have valuable implications against liver injury induced by environmental cadmium exposure.

## Data Availability

The original contributions presented in the study are included in the article/supplementary materials, further inquiries can be directed to the corresponding authors.
